# Multiple ways for the same destination: bone regeneration

**DOI:** 10.1186/s40902-022-00340-y

**Published:** 2022-03-02

**Authors:** Seong-Gon Kim

**Affiliations:** Department of Oral and Maxillofacial Surgery, College of Dentistry, Gangneung-Wonju National University, Gangneung, 25457 Republic of Korea

**Keywords:** Bone graft, Bone morphogenic protein-2, Osteoinduction, Osteoconduction, Hydroxyapatite

## Abstract

The regeneration of the bone is a challenging topic for maxillofacial plastic and reconstructive surgeons. For successful bone regeneration, timely providing of essential components is prerequisite. They are cellular components (osteoblasts, osteoclasts, and immune cells), extracellular matrix, and inorganic components (calcium and phosphate). Any deficient component can be provided from outside as a graft. Accordingly, there are many ways for successful bone regeneration. Selection of appropriate methods in an individualized situation is important.

## Background

Bone is the largest depot for calcium and phosphate. It is mainly responsible for the maintenance of blood calcium levels. The broken balance in blood calcium level is fatal. Bone remodeling is a coupled process between bone formation and resorption [1]. Physiologic bone remodeling is a continuous process. Broken balance during bone remodeling may result in either osteoporosis or osteopetrosis. Different types of hormones are involved in the bone remodeling process. Blood calcium levels can be elevated by bone resorption and decreased by excretion from the kidney [2].

In case of bone formation, inorganic components and organic components are required. Calcium and phosphate are essential inorganic components for bone formation [3]. Collagen is the main protein for the bone matrix. Bone formation is solely done by osteoblasts. Though osteoclast is mainly the responsible cell for bone resorption, the local acidic environment also accelerates decalcification and proteolysis of the bone matrix. Therefore, bone resorption is a less expensive process compared to bone formation in terms of energy requirement. Aging is an imbalance of energy consumption. Bone loss is much higher than bone gain during aging. Therefore, osteoporosis is a kind of aging process [4].

Except for physiological regulation of bone remodeling, some situations asked for active new bone formation. For example, fracture is a trauma in the bone. The bony defect between the fractured ends asks for active bone formation for repair. Surgical defect after treatment of bony lesion also requires active new bone formation [5]. If the size of the defect is small, the defect will be healed naturally without graft. In case of critical-sized defect, complete healing is not expected without a proper bone graft. Autogenous bone is usually taken in the operative theater and grafted immediately after being taken from the donor site. Cellular components in the autogenous bone are still alive for a couple of hours. Organic and inorganic fractions of bone are also intact. As it is autogenous, there is no immune reaction. Accordingly, autogenous bone graft has been considered as “gold standard” [1]. However, its amount is limited. In addition, donor site morbidity is another concern.

Several types of bone graft materials have been developed and available in the market. They have been classified as allograft, xenograft, and alloplast [1, 6]. This classification is originated from the source of the graft. It is not associated with the regenerative process. In addition, some osteoinductive materials such as bone morphogenic protein-2 (BMP-2) and platelet-derived growth factor (PDGF) are not included in this classification. Bone healing is also a staged process from acute inflammation to remodeling [6]. Different types of cells and resources are required in each step. Systemic condition of the patients may not be favorable for a smooth transition of each step. In that case, required graft materials should be tailored to each patient’s condition.

**Fig. 1:**
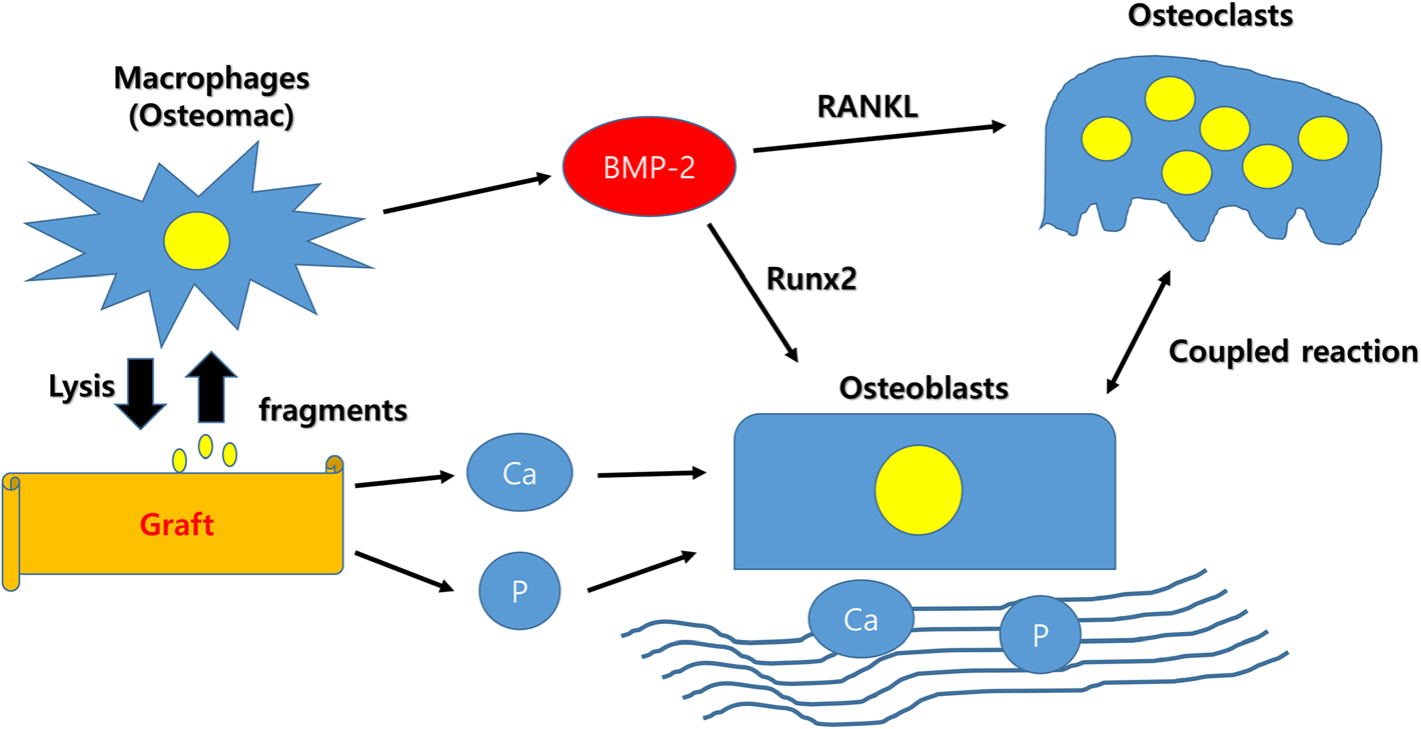
Schematic drawings of the bone regeneration process after grafting. As degrading graft by macrophages, degraded fragments may influence the macrophage. Some fragments stimulate macrophages to produce bone morphogenic protein-2 (BMP-2). It activates both osteoblast and osteoclast. Runx2, runt-related transcription factor-2; RANKL, receptor activator of nuclear factor kappa-B ligand

Unlike physiological remodeling, many immune cells such as macrophages are important cells for orchestrating the regenerative process [7]. However, it has been ignored largely. Immune cells are the main source for the production of osteoinductive factors. Therefore, bone regeneration should be controlled step by step. Calcium and phosphate should be supplied on demand. Matrix protein should be constructed with calcium phosphate deposition by osteoblasts. Angiogenesis is a prerequisite for the timely supply of amino acid and inorganic components to the bony defect. Macrophages should secrete osteoinductive factors such as BMP-2 for awakening resting osteoblasts. Bone grafts can help some processes among them. They may occupy the space to be filled with new bone, temporarily. In this case, the bone graft should allow creeping substitution. Otherwise, the graft may release osteoinductive factors and activate resting osteoblasts. Any new bone graft also should consider these processes (Fig. 1).

## Main text

### Calcium-based material

**Fig. 2:**
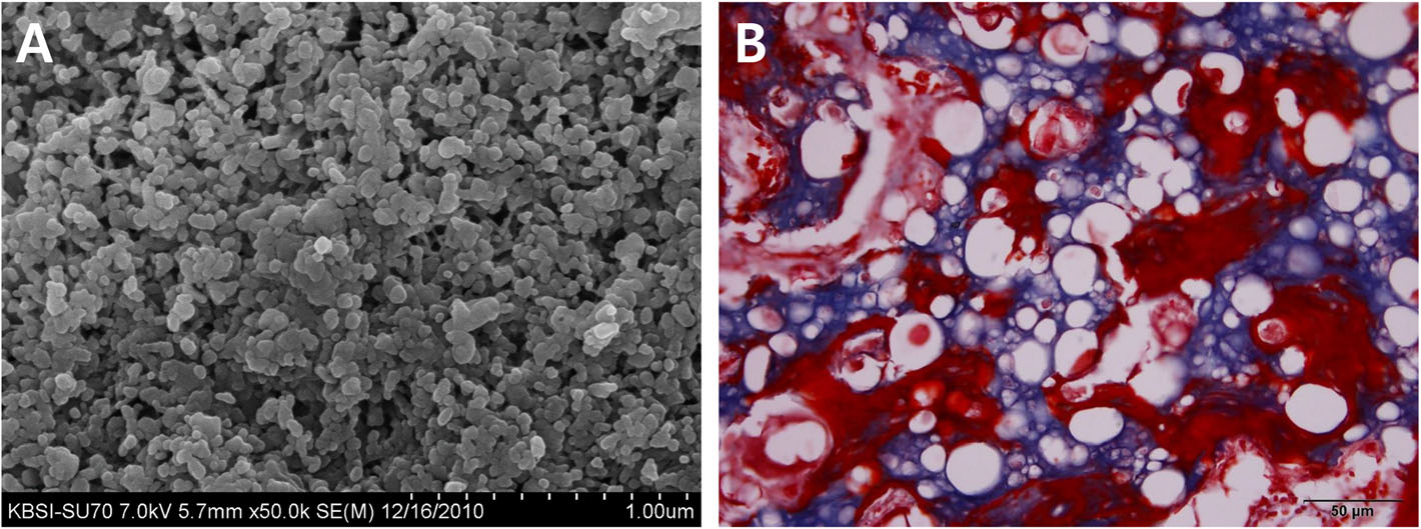
Hydroxyapatite (HA) as a bone graft. **A** Scanning microscopic image of HA crystal. **B** HA has been grafted into the rabbit’s calvarial defect. New bone formation is observed around the vacant spots at 8 weeks postoperatively. Vacant spots are occupied by HA and dissolved during the decalcification procedure

As bone is composed of calcium, calcium-based bone graft materials have been developed. If the graft is not autogenous, foreign proteins in the bone may cause immune reactions [8]. As foreign proteins are considered as an immunogen, these ingredients may induce severe inflammation. To avoid any unwanted immune response, deproteinization is an important step for processing animal or human-originated bone as a bone graft [9]. If bone matrix proteins are removed from the bone, only inorganic components will remain. Some synthetic materials such as hydroxyapatite (HA) or β-tricalcium phosphate (β-TCP) are bone grafts as calcium-based material (Fig. 2).

By experience, surgeons know a certain level of new bone formation is observed after grafting these materials. The mechanism of new bone formation in this type of material is still under investigation. Among them, calcium-based material increases local calcium concentration [10]. This situation is similar to bone resorption during physiological bone remodeling. When bone resorption is detected, osteoblasts will be activated. Immature bone is formed by osteoblasts. Subsequently, osteoblast-osteoclast coupled reaction will mature the bone as lamella bone [11].

This scenario is largely dependent on the degradation velocity of the graft. If grafts degrade too fast, local calcium concentration is exceeded the required range of calcium for bone regeneration. Too high calcium is toxic to many types of cells [12]. If the graft is not degraded, then released calcium will be absent. Both cases are improper for bone regeneration. Thus, the performance of these grafts is dependent on their degradation velocity. HA is known to be a slowly degraded material [13]. However, β-TCP is a fast degrading material. Accordingly, many types of natural bone are a combination of HA/ β-TCP and synthetic bone graft also mimics them [14].

### Scaffold

The component for improving bone matrix protein production is mainly focused on accelerating the production of type I collagen. This type of material is mainly used as a scaffold for bone graft [15]. They are acellular collagen [15], gelatin [16], and silk fibroin [17]. Bio-degradable synthetic polymers are also used as a scaffold. They are poly-l-lactic acid [18], polycaprolactone (PCL) [19], polyglycolic acid (PGA) [20], etc. They are known to accelerate the production of bone matrix protein. Accelerating the production of bone matrix protein is achieved by their degradation product (Fig. 1). As acellular collagen, gelatin, and silk fibroin are matrix proteins, they can be digested by proteolytic enzymes such as matrix metalloproteinases (MMPs) [21]. The degradation products are mainly peptides or amino acids. They may stimulate fibroblasts or osteoblasts to produce matrix proteins. Low-molecular weight silk fibroin accelerates type I collagen synthesis in osteoblast-like cells [22].

However, the scaffold cannot be used for repairing bony defects without any additive such as calcium-based material or osteo-inducers for angiogenesis and osteoinduction. In this case, the scaffold may be considered as a drug carrier. For optimal bone regeneration, controlled release from the scaffold is important [23]. When the scaffold is used solely, the bony defect may be mainly healed by fibrotic tissue. Because of these reasons, the calcium-based inorganic component has been frequently incorporated into the scaffold [8, 24].

Interestingly, BMP-2-soaked gelatin sponge has improved new bone formation [25]. In this case, gelatin sponge is used as a BMP-2 carrier for controlled release. Many types of osteo-inducers are hydrophilic and have low molecular weight. When this type of material is grafted into the bony defect without a proper carrier, it will be washed out. In humans, osteogenesis is started 3 or 4 weeks later event [26], and osteogenesis is maintained to 8 to 12 weeks later the event [26, 27]. The therapeutic concentration of multi-functional materials should be maintained at least 3 weeks and ideally until 12 weeks. Unfortunately, most drug carrier releases osteo-inducer more than 80% within the first week. Thereafter, a high dose of osteo-inducer is loaded initially considering unwanted loss and may increase the chance of ectopic bone formation. Overloading of some osteo-inducers may show complications. For example, BMP is usually loaded to collagen at several tens to hundreds milligram levels [28]. The effective dosage of BMP at the cell level is nano-gram level. In clinical practice, BMP-2 has been approved for maxillofacial reconstruction in 2007 [29]. Some patients receiving BMP have shown excessive swelling and pain as complications [30].

### Osteo-inducer (materials for angiogenesis and osteoinduction)

#### BMP

**Fig. 3:**
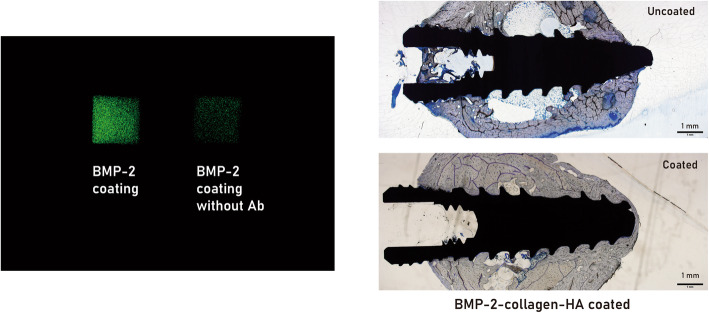
Bone morphogenic protein-2 (BMP-2) has been coated on a hydroxyapatite (HA)-coated titanium surface. Successful incorporation is confirmed by immunofluorescence image (left). When applied anti-BMP-2 antibody with green fluorescence, BMP-2 coated plate showed high signal intensity. However, the same kind of plate without application of anti-BMP-2 antibody showed low signal intensity. BMP-2, collagen, and HA combination-coated dental implant shows better peri-implant bone formation compared to the uncoated dental implant (right)

BMP is a representative material for osteo-inducer (Fig. 3). BMP is a family of transforming growth factor-beta [31]. There are several subtypes of BMPs. Among them, BMP-2, BMP-4, and BMP-7 have been considered as osteo-inducers. BMP-2 has been approved by the Food and Drug Administration (FDA) in 2008 and used for clinical practice [29, 30]. As a commercial product, BMP is provided as a recombinant protein. It can be produced either by *Escherichia coli* or Chinese hamster ovary (CHO) cells. The difference between them is the presence of post-translational modification.

BMP-2 induces osteogenesis via upregulating runt-related transcription factor-2 (Runx2) [32]. However, the cellular effect of BMP-2 is different from cellular types. BMP-2 can increase interleukins (ILs) and tumor necrosis factor-α (TNF-α) in immune cells [33]. BMP-2 administration to osteoclast can activate osteoclast via the nuclear factor-κB (NF-κB) pathway [34]. BMP-2 may promote fat formation via activation of peroxisome proliferator-activated receptor gamma (PPARγ) signaling [35].

The FDA-approved dosage of BMP-2 for humans is 1.5 mg/mL [30]. The therapeutic effect of BMP-2 is dose-dependent [36]. As mentioned above, a high dosage of BMP-2 has been administered to get the clinical result, and this has been the main reason for complications [30]. Excessive BMP-2 may diffuse to the adjacent area and increase the chance of ectopic bone formation [37]. Inflammatory complications such as seroma are frequently observed at the 1st week postoperatively [38, 39]. This type of inflammatory swelling can be life-threatening in the cervical area [40].

BMP is a required proper scaffold for successful bone regeneration. The scaffold for BMP can be used for other osteo-inducers. However, the optimal scaffold for each osteo-inducer is dependent on its physico-chemical feature. Most published papers used acellular collagen as a BMP scaffold. However, collagen is poor in BMP-2 retention capacity and may result in ectopic bone formation [30, 37]. Compared to collagen, hyaluronic acid and chitosan are better in retention capacity [41, 42]. Synthetic materials such as poly lactic-co-glycolic acid (PLGA) [43] or PCL [44] are also used for BMP scaffolds. Unlike collagen, synthetic materials degrade by hydrolysis. Therefore, its degradation velocity is assumed more predictable compared to the natural matrix. However, this is a hypothesis and not enough evidence in the clinical practice. Unlike laboratory conditions, the velocity of hydrolysis may be unpredictable in the body.

As direct incorporation of BMP-2 into the scaffold has several problems as mentioned above, indirect methods such as incorporating BMP-2 inducer into the scaffold can be considered. Many materials inducing inflammation can increase the expression level of BMP-2 in the macrophage [45, 46]. Lipopolysaccharide (LPS) is a bacterial endotoxin [47]. The administration of LPS on the periodontal ligament stem cells can increase the expression level of BMP-2 [47]. However, LPS induces too strong inflammation and may not be a proper agent for bone formation. Among foreign materials, silk sericin induces mild inflammation. Sericin can increase the expression level of BMP-2 in the macrophage [48]. The gelatin sponge incorporated with sericin can increase new bone formation compared to the gelatin sponge only graft [48, 49].

#### PDGF and vascular endothelial growth factor (VEGF)

Angiogenesis is a vital step for osteogenesis [30]. Medication-related jaw bone necrosis is a drug complication induced by impaired angiogenesis [50]. Diabetic bony lesion has also shown poor vascularity [49]. PDGF is rich in platelets. Concentrated PDGF can be produced by a simple centrifuge of whole blood [51]. They are platelet-rich plasma (PRP) or platelet-rich fibrin (PRF). PRP or PRF is a combination of growth factors and matrix protein [52]. Therefore, it can be used for small-sized defects without additional graft [53]. For the repair of large-sized defects, combination with calcium-based materials [54] or matrix protein [51, 53] has been tested and used.

As a recombinant protein, PDFG and VEGF have been tested for bone regeneration. VEGF only does not improve fracture healing, but VEGF plus BMP-2 enhances bone healing [55]. For the repair of tooth extraction socket, 0.3 mg/mL rhPDGF-BB plus calcium-based material is effective in clinical practice [56]. Compared to β-TCP alone, rhPDGF-BB plus β-TCP shows improved bone regeneration in the peri-implant defect [57].

#### Macrophage polarizing agent

**Fig. 4:**
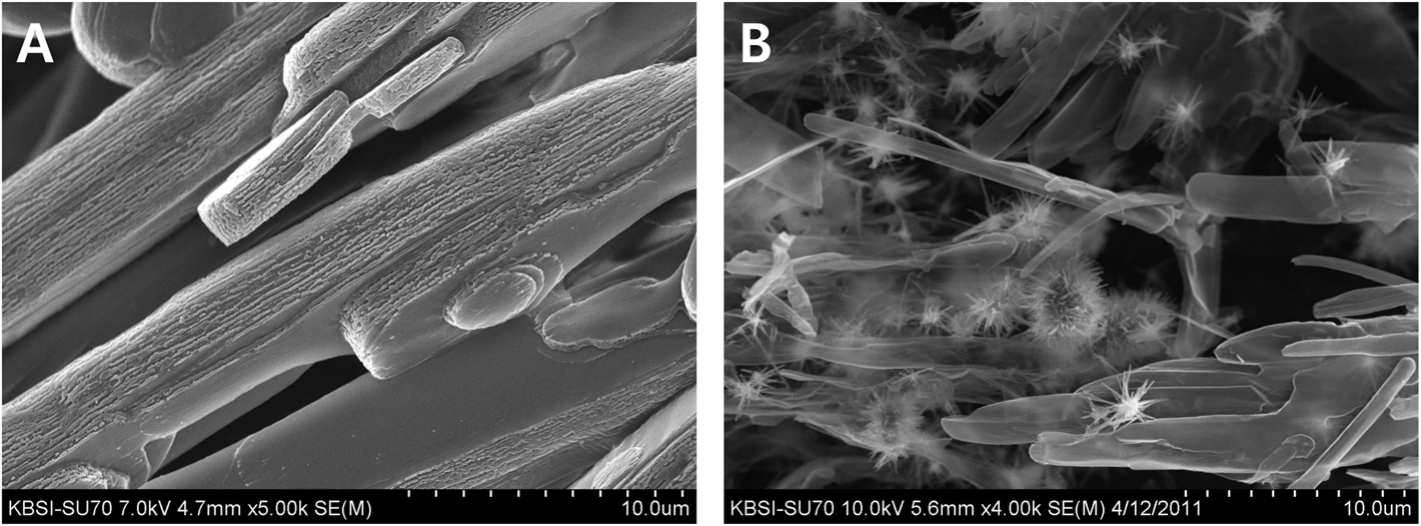
Scanning microscopic image of the porcine scapula (**A**) and 4-hexylresorcinol incorporated into the scapula (**B**). 4-Hexylresorcinol-incorporated scapula shows crystallization

**Fig. 5:**
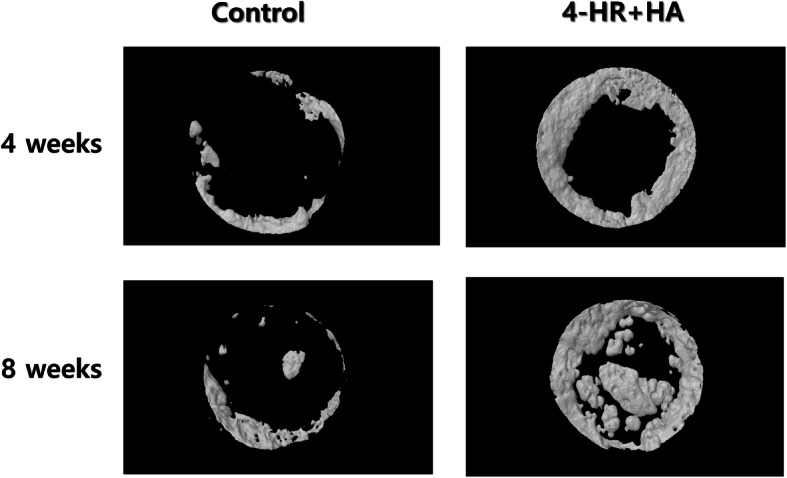
4-Hexylresorcinol incorporated into hydroxyapatite (HA) shows better bone formation compared to unfilled control in the critical-sized defect

Bone resident macrophage is called osteomac [58, 59]. Osteomac detects bone defects and orchestrated regeneration process [58]. During the acute inflammatory phase, osteomac recruits immune cells to defend microbial invasion and tissue destruction [59]. Osteomac invites fibroblasts and osteoblasts in the subsequent proliferating phase and remodeling phase [60]. When macrophage is classified as M0, M1, and M2, M1 type macrophage is prevalent in the acute inflammatory phase [7]. M2 type macrophage is prevalent in proliferating and remodeling phases [7]. Therefore, teaching macrophage to direct bone healing will be an interesting strategy for bone tissue engineering [7]. 4-Hexylresorcinol is identified as an M2 polarizing agent [61]. When 4-hexylresorcinol is incorporated into porcine scapula, dissolved calcium from the bone surface forms a crystal with 4-hexylresorinol (Fig. 4). When HA incorporated with 4-hexylresorinol is grafted into the critical-sized defects, enhanced new bone formation is observed (Fig. 5). Silk fibroin combined with 4-hexylresorcinol reduces foreign body reactions induced by silk protein and increases new bone formation [8].

#### Histone deacetylase (HDAC) inhibitor

HDAC inhibitor is an emerging osteo-inducer. HDAC regulates bone remodeling. Accordingly, HDAC influences not only osteogenesis, but also osteoclastogenesis [62]. However, chemical or genetic blockade of HDAC results in osteogenic differentiation [63]. Therefore, the balance between bone formation and bone resorption may be tilted to bone formation by HDAC inhibition. HDAC inhibitor increases the expression of Sp1. Sp1 is a transcription factor for the transforming growth factor-beta (TGF-β) family such as TGF-β1 and BMP-2/4. In addition, HDAC inhibitor increases the expression of Runx2. Runx2 is also a transcription factor, and its increased expression is associated with new bone formation [32]. Several papers have been published on this issue [64, 65]. HDAC inhibitors such as valproic acid (VPA) and trichostatin A (TSA) increase the expression level of BMP-2/4, osteocalcin, and Runx2 in dental pulp cells [66]. When a human mesenchymal stem cell is treated by 5-azacytidine plus TSA, the expression level of Runx2 is increased [67].

## Conclusion

Crabs and insects have their hard tissues on the outside. The main role of this tissue is protection. This hard tissue is introduced into the body in the vertebrate. There are many advantages of this evolution. The main advantage may be easy repair. In this review, there have been many trials for helping bone repair. Although many kinds of bone substitutes are available, the ideal bone graft is still elusive.

## Data Availability

Data sharing is not applicable to this article since no dataset was generated or analyzed during the current study.

## Abbreviations

BMP-2: Bone morphogenic protein-2
CHO: Chinese hamster ovary
FDA: Food and Drug Administration
HA: Hydroxyapatite
HDAC: Histone deacetylase
ILs: Interleukins
NF-κB: Nuclear factor-κB
PDGF: Platelet-derived growth factor
PLGA: Poly lactic-co-glycolic acid
PPARγ: Proliferator-activated receptor gamma
PRF: Platelet-rich fibrin
PRP: Platelet-rich plasma
TGF-β: Transforming growth factor-beta
TNF-α: Tumor necrosis factor-α
TSA: Trichostatin A
VEGF: Vascular endothelial growth factor
VPA: Valproic acid
β-TCP: β-Tricalcium phosphate
